# Characterizing the Use and Potential of Social Media for Education and Advancement in Emergency Medical Services (EMS)

**DOI:** 10.7759/cureus.77027

**Published:** 2025-01-06

**Authors:** Julianne Cyr, Emily Hutchens, Madison Rivard, Ashish Panchal, Rebecca Cash, Alexander Requarth, Jane Brice

**Affiliations:** 1 Emergency Medicine, University of North Carolina at Chapel Hill, Chapel Hill, USA; 2 Emergency Medicine, Massachusetts College of Art and Design, Boston, USA; 3 Emergency Medicine, The Ohio State University Wexner Medical Center, Columbus, USA; 4 Emergency Medicine, Massachusetts General Hospital, Boston, USA; 5 Emergency Medicine, University of North Carolina School of Medicine, Chapel Hill, USA

**Keywords:** education, emergency medical services, prehospital care, social media, social networking

## Abstract

Objective: Social media has significantly impacted how healthcare professionals access health education, news, and communication. However, Emergency Medical Services (EMS) clinicians’ professional use of social media remains unclear. Our objective was to characterize current EMS social media use and explore the potential for future professional use.

Methods: This was a cross-sectional survey of nationally-certified EMS clinicians. Data were collected from November to December 2018. Respondents were surveyed regarding demographics, social media use, and use of and preferences for accessing EMS-specific social media content. Descriptive statistics were calculated.

Results: A total of 3,087 participants responded to the survey. Of these, 2,705 respondents met the inclusion criteria. The majority of the sample were male (68%, n=1,838), non-Hispanic White (85%, n=2,200), with a mean (SD) age of 37 (±13.1) years. More than half (57%, n=1,531) were certified as EMTs and worked full-time (61%, n=1,387) in urban settings (60%, n=1,630). The most commonly used social media platforms included Facebook™ (80%, n=2,175) and Instagram™ (48%, n=1,285), with 66% (n=1,600) of respondents checking social media platforms several times per day. The most common professional uses of social media were for education (64%) and news (65%). Respondents reported interest in using social media for EMS resources, including education (83%, n=1,989), news/events (60%, n=1,439), products/innovations (57%, n=1,352), and networking/job opportunities (54%, n=1,285). Many EMS clinicians reported their EMS agency had a social media policy (63%), but 15% reported they were not allowed to use social media for professional activities.

Conclusions: EMS clinicians access social media frequently and express interest in its use for professional resources. However, many EMS do not currently use social media for professional activities. Considering EMS clinicians’ interest in social media for professional reasons and the growing use of social media across numerous professional fields, EMS agencies and organizations should consider broadening outreach to clinicians through social media.

## Introduction

Social media is a distinct form of media that, in recent decades, has changed the landscape of social interactions and the spread of information [1,2]. Merriam-Webster defines social media as "forms of electronic communication (such as websites for social networking and microblogging) through which users create online communities to share information, ideas, personal messages, and other content (such as videos)." As such, the definition of "social media" allows for a wide range of electronic-based sites or applications that might be included in its definition. The majority of Americans engage with social media daily, and many are using a variety of platforms [1]. Social media platforms vary in design and functionality, resulting in wide demographic differences in use. For example, professionals often use LinkedIn™ for career-related activities, and Snapchat™ is more often used by young people ages 18 to 24 years. While some social media platforms, such as YouTube™ and Facebook™, are popular with American adults, social media use in general is skewed heavily toward younger populations.

Not only are the majority of Americans engaging with social media daily, but these platforms are quickly rising as leading sources of news. More than 80% of adults reported receiving their news online in some form, with 18% reporting that social media is their sole source of news [2]. One-third of young adults ages 18 to 29 reported frequently getting their news from social media sites. Building on this trend, numerous professional medical fields have begun to use social media as a tool for education, networking, communication, and collaboration [3-5], both internally for members and externally with the public [6]. Eighty percent of medical students and as many as 97% of emergency medicine residents purposefully use social media for educational resources [7,8]. Not only are these materials sought for supplemental education, but medical students often perform better when using these resources [9]. Additionally, many public service fields have used social media as a tool to communicate with the public. For example, law enforcement and emergency management have successfully navigated social media to interact with and inform the public [10-13].

As Emergency Medical Services (EMS) is the intersection between the healthcare community and the wider public, there is a unique opportunity to leverage social media as a tool to improve the overall provision of patient care, especially with younger demographics [1]. With an average age of 35 years [14], EMS clinicians are typically a younger demographic than most other medical professionals [15]. Further, EMS agencies and organizations have the potential to build important community capital through social media engagement and to engage clinicians within their region or state. Additionally, individual EMS clinicians can access and utilize educational resources via social media for a more interactive and active educational opportunity rather than a passive textbook or lecture-based educational experience.

Free and open access to medical education (FOAM) is the free availability of educational resources that span multiple different medical topics. Previous research on EMS use of social media for FOAM focused on medical director members of a national emergency medicine organization and their agencies with limited exploration of social media platforms and preferences [16]. Our objective was to characterize current national EMS social media use and explore the potential for professional use of social media.

## Materials and methods

Study design and population

This was a cross-sectional evaluation of nationally certified EMS clinicians included in the National Registry of Emergency Medical Technicians (“National Registry”) database in the United States who responded to a survey on social media use through email. Though a national dataset of EMS clinicians does not exist, the National Registry maintains the largest database of EMS clinicians with over 400,000 people [17].

A sample size calculation was conducted assuming a three percent margin of error and a conservative 50/50 split in responses. It was determined that 1,067 responses from EMS clinicians would be needed to make estimates with 95% confidence. Based on past surveys among this population, the calculated sample size was inflated, assuming a conservative 12% response rate. We further inflated the sample to obtain enough responses in subgroups of interest (e.g., EMS clinicians aged 40 years or older), leading to a simple random sample of 35,000 EMS clinicians drawn from the National Registry’s database.

The survey utilized validated demographic and EMS-related items from the Longitudinal EMT Attributes and Demographics Study (LEADS), which have been used in large demographic descriptions of the EMS population [18]. Social media use survey questions were developed by the research team. Several survey questions regarding social media use were cognitively tested with prehospital providers prior to launching the survey. Modifications were made to social media survey questions using cognitive debriefing participants.

An email containing the link to the electronic questionnaire was sent to a random sample of EMS clinicians. After the initial release, follow-up reminders were sent at one- and two-week intervals from the original following a tailored Dillman method [19]. The survey contained 35 questions pertaining to social media use and demographics. The survey was estimated to take 10 minutes or less to complete. Data were collected from November 2, 2018, to December 13, 2018, within the NREMT’s annual member survey.

The American Institutes for Research Institutional Review Board and the University of North Carolina at Chapel Hill Institutional Review Board approved this project and granted signed waivers of consent.

Measurements and analysis

Included in the study were all respondents who were certified at the emergency medical technician (EMT) level or higher and were employed in at least one civilian EMS organization at the time of the survey. Outcomes of interest included current personal and professional social media use, EMS agency social media policies, and interest in social media use for professional reasons. Responses to the social media survey were linked to respondents’ demographics located in the National Registry’s EMS database.

Dichotomous variables were created for minority status (i.e., non-Hispanic White or minority), urbanicity (rural (<25,000 residents) or urban (≥25,000 residents)), and number of EMS agency affiliations (1 or ≥2). Age was categorized into quartiles. For the purposes of describing the sample, the national certification level was defined as EMT, advanced EMT (AEMT)/EMT-intermediate (EMT-I), or paramedic. These certifications were dichotomized for further social media use analyses into basic life support (BLS), which consisted of EMTs, and advanced life support (ALS), which consisted of EMT-Is, AEMTs, and paramedics. Employment status was defined as full-time, part-time, or volunteer. Years of EMS experience were collapsed into ≤2 years, 3-10 years, and ≥11 years. Descriptive statistics were calculated on demographics, social media use, interest, and EMS agency social media policy using STATA IC version 14.2 (StataCorp LP, College Station, USA).

## Results

Of the 34,956 EMS clinicians emailed, 35.7% opened the survey link, 1.1% (n=395) partially completed the survey, and 8.8% (n=3,087) clinicians completed the survey. As per the inclusion criteria, the final analysis population was 2,705 respondents. 

Respondent demographics were similar to those of nationally certified EMS personnel (Table 1) [14, 20, 21]. The majority of the sample were non-minority (85.2%; n=2,200), males (68.0%;n=1,838), working full-time (62.0%; n=1,387) in urban EMS agencies (60.3%; n=1,630). Respondents’ mean age was 37 (standard deviation: +/-13.1) years. Most respondents were nationally certified at the EMT level (56.6%; n=1,531). 

**Table 1:** Demographics of survey respondents (n=2,705) EMS: Emergency Medical Services

	number (percent)
Sex	
Male	1,838 (68.0)
Female	860 (32.0)
Missing	7 (--)
Age Quartiles (years)	
18-25	514 (20.7)
26-35	627 (25.2)
36-47	694 (27.9)
48-80	650 (26.2)
Missing	220 (--)
Minority Status	
Non-Hispanic White	2,200 (85.2)
Minority	381 (14.8)
Missing	124 (--)
Urbanicity of Primary EMS System (total population)	
Urban (≥25,000 residents)	1,630 (60.3)
Rural (<25,000 residents)	1,075 (39.7)
National Certification Level	
EMT	1,531 (56.6)
AEMT / EMT-I	147 (5.4)
Paramedic	1,027 (38.0)
Employment Status	
Full-time	1,387 (62.0)
Part-time	308 (13.8)
Volunteer	543 (24.3)
Missing	467 (--)
EMS Organization Affiliations	
1	1,805 (66.8)
2+	899 (33.2)
Missing	1 (--)
Years of EMS Experience	
≤2 years	868 (32.1)
3-10 years	780 (28.8)
≥11 years	1,057 (39.1)

Social media use was assessed within the survey. Most EMS clinicians (97.5%; n= 2,381) engaged in at least some social media use. The majority engaged with social media at least daily (83.8%; n= 2,047). Social media use predominated across all EMS clinician certification levels, age quartiles, and urbanicities; very few EMS clinicians used social media almost never or never (2.5%; n=62) (Table 2). 

**Table 2:** Frequency of social media use by respondent characteristics EMS: Emergency Medical Services

	Social Media Usage				
	Several times a day number (percent)	Once a day number (percent)	Several times a week number (percent)	Several times a month number (percent)	Almost never or never number (percent)
Certification Level					
BLS	924 (66.4)	251 (18.0)	140 (10.0)	46 (3.3)	30 (2.2)
ALS	676 (64.3)	196 (18.6)	100 (9.5)	48 (4.6)	32 (3.0)
Age Quartiles (years)					
18-25	407 (80.8)	66 (13.1)	23 (4.6)	4 (0.8)	4 (0.8)
26-35	421 (72.8)	87 (15.1)	50 (8.7)	10 (1.7)	10 (1.7)
36-47	385 (62.5)	122 (19.8)	69 (11.2)	27 (4.4)	13 (2.1)
48-80	267 (48.6)	128 (23.3)	82 (14.9)	43 (7.8)	29 (5.3)
Urbanicity of Primary EMS System (total population)					
Urban (≥25,000 residents)	1,000 (67.6)	258 (17.4)	132 (8.9)	51 (3.4)	39 (2.6)
Rural (<25,000 residents)	600 (62.3)	189 (19.6)	108 (11.2)	43 (4.5)	23 (2.4)
Total	1,600 (65.5)	447 (18.3)	240 (9.8)	94 (3.8)	62 (2.5)

The most common social media platforms used among all respondents were Facebook™ (80.4%; n= 2,175), Instagram™ (47.5%; n= 1,285), Snapchat™ (41.8%; n=1,131), and YouTube™ (37.3%; n= 1,009) (Table 3). This trend was also noted among daily users with 90.7% (n=1,872) using Facebook™, 57% (n=1,175) using Instagram™, 50.8% (n=1,047) using Snapchat™, and 42.5% (n=876) using YouTube™ (results not shown). 

**Table 3:** Social media platform use by respondent characteristics EMS: Emergency Medical Services

	Facebook™ number (percent)	Instagram™ number (percent)	Snapchat™ number (percent)	YouTube™ number (percent)	LinkedIn™ number (percent)	X™ (formerly Twitter™ number (percent)	Google+™ number (percent)	Other (e.g., MySpace™, Periscope™) number (percent)	None number (percent)
Certification Level									
BLS	1,231 (80.4)	788 (51.5)	732 (47.8)	597 (39.0)	328 (21.4)	321 (21.0)	194 (12.7)	37 (2.4)	138 (9.0)
ALS	944 (80.4)	497 (42.3)	399 (34.0)	412 (35.1)	309 (26.3)	262 (22.3)	141 (12.0)	30 (2.6)	115 (9.8)
Age Quartiles (years)									
18-25	447 (87.0)	396 (77.0)	408 (79.4)	269 (52.3)	96 (18.7)	161 (31.3)	58 (11.3)	12 (2.3)	10 (2.0)
26-35	505 (80.5)	352 (56.1)	318 (50.7)	239 (38.1)	141 (22.5)	118 (18.8)	72 (11.5)	16 (2.6)	44 (7.0)
36-47	572 (82.4)	284 (40.9)	232 (33.4)	227 (32.7)	182 (26.2)	161 (23.2)	83 (12.0)	21 (3.0)	75 (10.8)
48-80	484 (74.5)	143 (22.0)	87 (13.4)	192 (29.5)	180 (27.7)	103 (15.8)	96 (14.8)	12 (1.8)	100 (15.4)
Urbanicity of Primary EMS System (total population)									
Urban (>25,000)	1,285 (78.8)	828 (50.8)	688 (42.2)	636 (39.0)	390 (23.9)	366 (22.5)	185 (11.3)	44 (2.7)	148 (9.1)
Rural (<25,000)	890 (82.8)	457 (42.5)	443 (41.2)	373 (34.7)	247 (23.0)	217 (20.2)	150 (14.0)	23 (2.1)	105 (9.8)
Total	2,175 (80.4)	1,285 (47.5)	1,131 (41.8)	1,009 (37.3)	637 (23.5)	583 (21.6)	335 (12.4)	67 (2.5)	253 (9.4)

Social media use for EMS-related content was also evaluated. Respondents were asked how many days in the two weeks prior to the survey they used social media to access EMS education, EMS news/events, EMS products/innovations, and EMS networking/job opportunities. The median social media use for any EMS-related content was 1 (IQR 0-3) day during the two-week period. The detailed use by EMS clinicians across the four categories is described in Table 4.

**Table 4:** Frequency (days) of respondents’ use of emergency medical service content on social media in the past two weeks EMS: Emergency Medical Services

Topic	Number of days in past 2 weeks	Number (percent)	Median (IQR) days
EMS education	0 days	827 (35.6)	1 (0-3)
	1 day	457 (19.7)	
	2-3 days	528 (22.7)	
	4-14 days	509 (21.9)	
EMS news/events	0 days	789 (34.6)	1 (0-3)
	1 day	421 (18.5)	
	2-3 days	503 (22.1)	
	4-14 days	567 (24.9)	
EMS products/innovations	0 days	1,101 (49.1)	1 (0-2)
	1 day	412 (18.4)	
	2-3 days	367 (16.4)	
	4-14 days	361 (16.1)	
EMS networking/job opportunities	0 days	1,274 (56.5)	0 (0-2)
	1 days	330 (14.6)	
	2-3 days	298 (13.2)	
	4-14 days	354 (15.7)	

Respondents were also asked which types of EMS content they would be most interested in accessing on social media if given the opportunity (Table 5). Respondents reported they were most interested in accessing EMS education (83.1%; n= 1,989). Additionally, EMS clinicians were asked if they used social media to engage in online journal clubs or to access FOAM education. Largely, EMS clinicians did not participate in online journal clubs (80.0%; n=1,887) and were unaware of FOAM education (89.8%; n= 2,331) (results not shown).

**Table 5:** Respondents’ interests in using social media for professional reasons EMS: Emergency Medical Services

	Number (percent)
EMS education	1,989 (83.1)
Educational and procedural videos	1,921 (81.0)
Research Articles	1,205 (50.8)
Quick reference materials	1,192 (50.2)
Case reports	1,143 (48.2)
Blogs	289 (12.2)
Other	43 (1.8)
EMS news/events	1,439 (60.1)
EMS products/innovations	1,352 (56.5)
EMS networking/job opportunities	1,285 (53.7)

In recognition of the variable use of social media in personal versus professional spaces, respondents were asked about social media policy and use within their EMS agencies. Of all respondents, 1,704 (63.0%) confirmed their main EMS agency had an official social media policy, while 505 (18.7%) did not know of a social media policy (results not shown). Of those respondents with a social media policy, 16.6% (n=284) were prohibited from using social media while on duty, and 44.4% (n=756) were prohibited from posting comments or pictures about their EMS agency. Further, 56.2% (n=958) believed that their social media use may be monitored by their EMS agency. 

## Discussion

The purpose of this study was to characterize EMS's social media use and explore the potential for professional use of social media. The majority of EMS clinicians, despite age, urbanicity, or certification level, were at least daily users of social media. While Facebook™ is not typically considered a central FOAMed platform, it was included due to its prominence among social media platforms at the time of this survey. EMS clinicians utilized a variety of social media platforms, with Facebook™ as the most preferred. These trends reflect the national preference for Facebook™ as one of the most popular social media platforms [1], but the most popular platform among Americans, YouTube™, was reportedly used with far less frequency among EMS. Of note, EMS appeared to use more “professional” social media platforms, such as LinkedIn™ and X™ (formerly Twitter™), at similar rates to that of adult Americans but consumed more casual or personal social media platforms, such as Snapchat™ and Instagram™, more frequently than the general American population.

Despite research indicating limited knowledge of FOAM or the use of X™ (formerly Twitter™) or Facebook™ for FOAM [16], this study demonstrates a strong interest in using social media for professional education and networking. Participants reported interest in opportunities to engage with EMS educational content via social media, particularly in accessing procedural videos. These interests are reflected in similar fields that were early adopters of social media. Emergency medicine interns view social media as a valuable supplement to traditional learning because topics are naturally sorted and prioritized by their importance and value to the targeted learning community [8]. Additionally, more diverse uses of social media beyond education have been incorporated with success among other public service fields such as policing and emergency management [10-13]. With EMS as the intersection between public service and medicine, and with a growing presence of both communities on social media, the use of social media by EMS for professional endeavors appears to be a natural step for the field.

This study further demonstrates a disconnect between the types of information EMS clinicians are currently accessing via social media and what their stated interests are. Although a large proportion of clinicians reported interest in accessing EMS education via social media, only a small proportion actually accessed these materials. These trends are further demonstrated by differences between the use and interest in accessing EMS networking opportunities. The reason for this difference is unclear and may be multifactorial, including lack of time to access, lack of appropriate platforms to access, lack of available resources in existence, or even workplace policies. Many respondents reported strict social media guidelines from their agency that may restrict clinicians from using social media for professional purposes. For example, social media networking would be discouraged for clinicians whose agencies restrict personnel from associating their online presence with their EMS workplace. Future evaluations will need to explore the dissonance between current and preferred access to EMS content on social media platforms in more detail.

The importance of social media policies, either to maintain patient health information privacy or prevent the viral spread of inaccurate health and safety information via social media [12,20], cannot be minimized. However, these concerns can be combated by using carefully curated and vetted resources, including content designed by known educational entities such as the FOAM project [21,22], and resources developed by known field-specific organizations and journals. The ease of access must be balanced with peer review, ensuring that reliable information is disseminated. There are obvious benefits to an EMS social media professional presence (e.g., interactive educational content and community engagement). Social media is largely free and accessible on computers, tablets, and smartphones [1]. There is limited need for the same physical resources necessary for traditional educational engagement such as meeting space and transportation arrangements. Social capital can be promoted in these online communities [10], particularly through shared decision-making between professional and community stakeholders, public building of collaborative relationships, and the inherent transparency of these activities online. As previously stated, the advantages of social media use have been realized in various fields. Social media has proven to be a valuable resource in medical education. Medical schools have experienced improvement in students’ knowledge, attitudes, and skills, in addition to the promotion of learner engagement [9]. Online medical communities have been successfully developed and largely dominated by single-discipline communities, allowing members to “share domain knowledge” [5] and establish lines of communication with colleagues [20]. Police departments have seen some benefits through community engagement and information sharing [12]. EMS clinicians are interested in using social media to access more field-specific content; as such, EMS agencies and organizations should take the necessary steps to utilize social media for professional development and community building.

Limitations

Though this survey assessed social media use among EMS clinicians, some self-selection was evident to the research team. Several non-respondents emailed the National Registry after receiving the survey invitation to indicate they did not use social media and therefore would not be completing the survey. The research team made the purpose of the survey evident in the body of the email and the survey instructions by stating “The proposed survey will offer a snapshot of how EMS providers currently use or desire to use social media for their professional development and education. We hope to use this information to further understand how we can work with EMS providers in other regions to improve professional communication, education, and access to information.” In addition, not all EMS clinicians understood how social media was defined; some free text responses to the survey question “What social media platforms do you use?” included “email,” “newspaper,” and “cable tv.”

The response rate for this survey was 8.8% (n=3,807). Of the surveys emailed to EMS clinicians, 5.3% (n=2,705) both responded and met inclusion criteria. Though this appears on the surface to be a low response rate for a survey of nearly 35,000 nationally certified EMS clinicians, this response rate is similar to those received from other surveys delivered to the same population. In addition, the sample was generally representative of the National Registry population according to participant demographics, as compared to previous research [14,23,24]. However, the sample is not necessarily representative of the EMS clinician population across the US as national certification is not a mandatory criterion to practice as an EMS clinician in every EMS agency.

Finally, the availability of social media platforms is ever-evolving. At the time this survey was administered, the researchers included several common social media platforms as response options (i.e., Facebook™, Google+™, Instagram™, LinkedIn™, MySpace™, Periscope™, Snapchat™, X™ (formerly Twitter™), YouTube™), in addition to an “other” response option with a fill-in-the-blank response and an “I do not use social media at all” response option. The researchers could not possibly include all response options and therefore relied on EMS clinicians to self-identify and report any additional social media platforms (e.g., WhatsApp™, ResearchGate™). This reliance on participant identification and reporting of social media platforms not listed may have resulted in lower usage reported for these additional platforms. In the time since this survey was taken (2018) the social media landscape has continued to change, including a change in ownership of Twitter™, now X™, the increased prevalence of platforms such as TikTok™ and Instagram™ and questions of social media's ability to protect users private information.

## Conclusions

EMS professionals access a variety of social media platforms and express interest in using social media for professional reasons. However, there remains a challenge for EMS agencies to strike a balance between encouraging appropriate professional social media use and maintaining patient and agency privacy. Considering EMS's interest in using social media for education and engagement, EMS agencies and organizations should consider broadening outreach to their community through social media. Further exploration of EMS engagement across social media platforms should be assessed to determine, which if any, platforms meet the foremost needs and preferences for accessing the EMS content clinicians identified as most desirable.

## Figures and Tables

**Figure 1:**
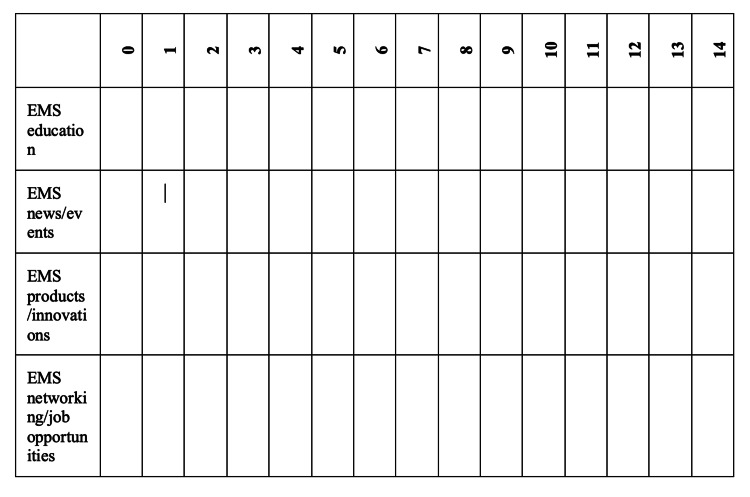
Question 21 EMS: Emergency Medical Services

## Appendices

Appendix A

Social Media Use in EMS

Page exit logic: Jump to #29 IF: ((#1 Question "At what level are you currently practicing as an EMS professional?" is one of the following answers ("None") OR #2 Question "How many years have you worked as an EMS professional?" is one of the following answers ("I have never worked as an EMS professional")) OR #3 Question "For how many different organizations do you currently perform EMS work?" is one of the following answers ("0"))

1) At what level are you currently practicing as an EMS professional?

( ) None

( ) Emergency Medical Responder

( ) Emergency Medical Technician

( ) Emergency Medical Technician - Intermediate [The Emergency Medical Technician - Intermediate level is only used in states in which individuals are licensed at this level.]

( ) Advanced Emergency Medical Technician

( ) Paramedic

( ) Other - Please specify: _________________________________________________

2) How many years have you worked as an EMS professional?

( ) I have never worked as an EMS professional

( ) Less than one year

( ) 1-2 years

( ) 3-4 years

( ) 5-7 years

( ) 8-10 years

( ) 11-15 years

( ) 16-20 years

( ) 21 or more years

3) For how many different organizations do you currently perform EMS work?

( ) 0

( ) 1

( ) 2 or more

4) Which of these best describes your employment status with your main EMS Agency?

( ) Full-time

( ) Part-time

( ) Volunteer

5) At what level do you currently function in your main EMS Agency?

( ) Emergency Medical Responder

( ) Emergency Medical Technician

( ) Emergency Medical Technician - Intermediate

( ) Advanced Emergency Medical Technician

( ) Paramedic

( ) Other - Please specify: _________________________________________________

6) How many years have you worked at your main EMS agency?

( ) Less than one year

( ) 1-2 years

( ) 3-4 years

( ) 5-7 years

( ) 8-10 years

( ) 11-15 years

( ) 16-20 years

( ) 21 or more years

7) Which of the following best describes your primary role at your main EMS job?

( ) Patient Care Provider [A person whose primary role is the provision of EMS services to patients.]

( ) Educator [A person whose primary role is instructing individuals enrolled in an approved or accredited EMS training course or providing continuing education required for maintenance of licensure.]

( ) Preceptor [A person whose primary role is training individuals enrolled in an approved or accredited EMS training course in a clinical setting.]

( ) Dispatcher/Call Taker [A person whose primary role is EMS communications.]

( ) Administrator/Manager [A person whose primary role is the management and direction of an organization providing EMS services.]

( ) First-line Supervisor [A person whose primary role is the direct supervision of individuals providing EMS services.]

( ) Other -  Please specify: _________________________________________________

8) Which of the following best describes your main EMS agency/organization?

( ) Hospital [Refers to EMS agencies that are under the direct control of a hospital, regardless of the type of organization that runs the hospital.]

( ) Fire Department [An organization from which fire and EMS services are provided, regardless of the type of organization that runs the Fire Department. Volunteer fire departments should be included here.]

( ) Tribal [Operated by a federally recognized Indian or Alaska Native Tribe.]

( ) Military [Operated by one of the U.S. Armed Forces and staffed by active duty personnel.]

( ) Government, Non-Fire Department [Operated directly by a federal, state, county, or local government entity other than the U.S. Armed Forces.]

( ) Private [Operated under the direct control of a for-profit or not-for-profit organization other than a hospital. Volunteer rescue squads that are operated independently of a fire department should be included here.]

( ) Air Medical [An organization which provides air ambulance services, regardless of the type of organization which runs the air ambulance service.]

( ) Other - Please specify: _________________________________________________

9) Which of the following best describes the primary type of service provided by your main EMS agency/organization?  If more than one type of service is provided, pick the service with the greatest number of calls in the past 12 months.

( ) Primarily 911 response with or without transport capability [Immediate response to an incident location, regardless of method of notification (for example, 911, direct dial, walk-in, flagging down).]

( ) Primarily medical transport (convalescent) [Transport of a patient from one health facility to another.]

( ) Equal mix of 911 and medical transport (convalescent)

( ) Clinical services [Provision of clinical services in an non-ambulance clinical setting such as emergency department, medical office, or dialysis clinic.]

( ) Mobile Integrated Healthcare & Community Paramedicine [Provision of clinical services in an out-of-hospital community setting.]

( ) Other - Please specify: _________________________________________________

Page exit logic: Skip to #12 IF: #10 Question "Does your main EMS agency have a policy about social media use?" is one of the following answers ("No", "I don't know")

10) Does your main EMS agency have a policy about social media use?

( ) Yes

( ) No

( ) I don't know

11) What does your main EMS agency's social media policy include? (Select all that apply)

( ) I am prohibited from using social media while on duty

( ) I am prohibited from posting comments or pictures about my EMS agency on social media

( ) My social media use may be monitored by my EMS agency

( ) I am prohibited from sharing Protected Health Information (PHI) such as patient name, address, photo, or details of a call

( ) I am prohibited from sharing proprietary or confidential information about my EMS agency

( ) I am prohibited from posting statements that are derogatory, vulgar, offensive, or abusive

( ) Other - Please specify: _________________________________________________

Page exit logic: Skip to #25 IF: #12 Question "What social media platforms do you use? (Select all that apply)" is one of the following answers ("I do not use social media at all")

12) What social media platforms do you use? (Select all that apply)

( ) Facebook

( ) Google+

( ) Instagram

( ) LinkedIn

( ) Myspace

( ) Periscope

( ) Snapchat

( ) Twitter

( ) YouTube

( ) Other - Please specify: _________________________________________________

( ) I do not use social media at all

13) Of the social media platforms you use, which one do you use the most?
 

_______________________________

14) How often do you check your most preferred social media platform?

( ) Several times a day

( ) Once a day

( ) Several times a week

( ) Several times a month

( ) Almost never or never

15) How often do you check any social media platform?

( ) Several times a day

( ) Once a day

( ) Several times a week

( ) Several times a month

( ) Almost never or never

16) On which device do you most often access social media platforms?

( ) Smart phone

( ) Tablet (iPad, Amazon Fire, Samsung Galaxy Tab, etc.)

( ) Laptop/Desktop computer

Page exit logic: Skip to #19 IF: #17 Question "Do you use social media for professional education purposes, such as learning more about EMS research, innovations, new products, or updates to protocols?" is one of the following answers ("No")

17) Do you use social media for professional education purposes, such as learning more about EMS research, innovations, new products, or updates to protocols?

( ) Yes

( ) No

18) Which social media platforms do you use for professional education purposes? (Select all that apply)

( ) Facebook

( ) Google+

( ) Instagram

( ) LinkedIn

( ) Myspace

( ) Snapchat

( ) Twitter

( ) YouTube

( ) Other - Please specify: _________________________________________________

Page exit logic: Skip to #21 IF: #19 Question "Do you use social media platforms for professional networking, such as meeting other EMS providers or finding out about EMS events?" is one of the following answers ("No")

19) Do you use social media platforms for professional networking, such as meeting other EMS providers or finding out about EMS events?

( ) Yes

( ) No

20) Which social media platforms do you use for professional networking purposes? (Select all that apply)

( ) Facebook

( ) Google+

( ) Instagram

( ) LinkedIn

( ) Myspace

( ) Snapchat

( ) Twitter

( ) YouTube

( ) Other - Please specify: _________________________________________________

21) How many days in the past two weeks have you used social media platforms to access information about:

22) If you had the opportunity, which of these would you be interested in accessing on social media? (Select all that apply)

( ) EMS education

( ) EMS news/events

( ) EMS products/innovations

( ) EMS networking/job opportunities

23) Which type of EMS education would you prefer to access via social media? (Select top three choices)

( ) Educational and procedural videos

( ) Blogs

( ) Research articles

( ) Case reports

( ) Quick reference material (e.g., an infographic)

( ) Other - Please specify: _________________________________________________

24) Have you ever joined a journal club or similar group on Twitter, Facebook, or another social media platform?
 

( ) Yes

( ) No

Page exit logic: Skip to #27 IF: #25 Question "Does your main EMS agency currently use a social media platform?" is one of the following answers ("No","I don't know")

25) Does your main EMS agency currently use a social media platform?

( ) Yes

( ) No

( ) I don't know

26) For what purpose does your main EMS agency use social media? (Select all that apply)
 

( ) Education

( ) Sharing local EMS news or events

( ) Sharing national EMS news or events

( ) Sharing funny/interesting stories or pictures

( ) Other - Please specify: _________________________________________________

27) Does your main EMS agency encourage use of social media for educational purposes (outside of required training)?
 

( ) Yes

( ) No

( ) I don't know

28) Do you know about the Free Open Access Medical (FOAM) education movement on social media to enhance medical knowledge and communication between providers?

( ) Yes, I have used/followed FOAM on social media

( ) Yes, I have heard about FOAM, but I have not used it

( ) No, I have never heard of FOAM

29) In what state do you live?

( ) Alabama

( ) Alaska

( ) Arizona

( ) Arkansas

( ) California

( ) Colorado

( ) Connecticut

( ) Delaware

( ) Florida

( ) Georgia

( ) Hawaii

( ) Idaho

( ) Illinois

( ) Indiana

( ) Iowa

( ) Kansas

( ) Kentucky

( ) Louisiana

( ) Maine

( ) Maryland

( ) Massachusetts

( ) Michigan

( ) Minnesota

( ) Mississippi

( ) Missouri

( ) Montana

( ) Nebraska

( ) Nevada

( ) New Hampshire

( ) New Jersey

( ) New Mexico

( ) New York

( ) North Carolina

( ) North Dakota

( ) Ohio

( ) Oklahoma

( ) Oregon

( ) Pennsylvania

( ) Rhode Island

( ) South Carolina

( ) South Dakota

( ) Tennessee

( ) Texas

( ) Utah

( ) Vermont

( ) Virginia

( ) Washington

( ) Washington, D.C.

( ) West Virginia

( ) Wisconsin

( ) Wyoming

30) Which of the following best describes the community in which you do most of your EMS work?

( ) Rural area (less than 2,500 people)

( ) Small town (2,500 - 24,999 people)

( ) Medium town (25,000 -74,999 people)

( ) Large town (75,000 - 149,999 people)

( ) Mid-sized city (less than 500,000 people)

( ) Suburb/fringe of a mid-sized city

( ) Large city (500,000 or more people)

( ) Suburb/fringe of a large city

31) In what year were you born?

_________________________________________________

32) What is the highest level of education you have completed?

( ) Didn't complete high school

( ) High school graduate/GED

( ) Some college

( ) Associate's Degree

( ) Bachelor's Degree

( ) Master's Degree

( ) Doctoral Degree

33) What is your sex?

( ) Male

( ) Female

34) Are you Hispanic or Latino?

( ) Yes

( ) No

( ) Prefer not to answer

35) Which of the following best describes you? You may choose more than one.

( ) American Indian or Alaskan Native

( ) Asian

( ) Black or African American

( ) Native Hawaiian or other Pacific Islander

( ) White

( ) Prefer not to answer
